# Beyond the Incision: A Comparative Study of Suprainguinal and Inguinal Varicocele Surgeries

**DOI:** 10.7759/cureus.67073

**Published:** 2024-08-17

**Authors:** Bhushan Shah, Jayant Bajaj, Adithya R Vijendra

**Affiliations:** 1 General Surgery, Dr. D. Y. Patil Medical College, Hospital and Research Centre, Dr. D. Y. Patil Vidyapeeth (Deemed to be University), Pune, IND

**Keywords:** infertility, semen analysis, inguinal approach, suprainguinal approach, varicocele

## Abstract

Background

Varicocele, characterised by the abnormal dilation of the pampiniform plexus of scrotal veins, is a prevalent and treatable cause contributing to male infertility, affecting 40% of men experiencing primary infertility and 80% of those with secondary infertility. Often asymptomatic, it can lead to chronic scrotal pain or a feeling of heaviness. Surgical interventions such as open, laparoscopic, or microsurgical varicocelectomy aim to eliminate venous reflux into the scrotum. This study sought to assess and evaluate the surgical outcomes and postoperative complications of the suprainguinal and inguinal approaches to varicocelectomy, offering evidence-based insights to improve varicocele management.

Methodology

A total of 60 males diagnosed with grade II or III unilateral or bilateral varicocele were included in the study. Patients with subclinical or grade I varicocele, recurrent varicocele, or concomitant inguinoscrotal pathology (hydrocele, epididymal cyst, and inguinal hernia) were excluded. Participants were randomly assigned to one of two groups based on the surgical technique: Group A (suprainguinal approach) and Group B (inguinal approach). Surgical outcomes were assessed by evaluating operating time, postoperative pain, wound hematoma, wound infection, hydrocele development, testicular atrophy, and semen analysis, both pre- and postoperatively.

Results

The study included 60 patients with a mean age of 29.05±5.96 years and an age range of 18-40 years. The suprainguinal approach offers a significantly shorter operating time than the inguinal approach (33.1 vs. 40.8 minutes). Both surgical techniques resulted in similar postoperative pain levels. The incidence of complications, such as wound hematoma, wound infection, and hydrocele development, showed no significant differences between the two approaches. In Group A (suprainguinal approach), the rates were 3.3%, 6.6%, and 3.3%, respectively, while in Group B (inguinal approach), they were 6.6%, 13.3%, and 6.6%. Additionally, 75% of patients in the infertility group showed improvements in semen parameters, with 80% in Group A and 71.4% in Group B, with no significant difference between the surgical approaches.

Conclusion

The suprainguinal and inguinal approaches to varicocelectomy effectively manage varicoceles, with the suprainguinal approach offering a shorter operating time. Postoperative complications and improvements in semen parameters were comparable between the two methods.

## Introduction

Varicocele is characterised by the abnormal dilation and enlargement of the pampiniform plexus of scrotal veins, resulting in venous blood stasis within the spermatic cord [1]. It is recognised as the most common treatable and surgically correctable condition contributing to male infertility, affecting 40% of men experiencing primary infertility and 80% of those with secondary infertility [2]. The predominance of left-sided varicoceles, accounting for approximately 85-90% of cases, is due to the extended course of the left internal spermatic vein and its perpendicular entry into the left renal vein [2]. Varicoceles are often asymptomatic and discovered during routine exams or infertility evaluations. Symptoms, when present, include chronic scrotal pain or heaviness, worsening with prolonged standing or strenuous activity [3]. A characteristic "bag of worms" appearance is observed during the clinical examination of large varicoceles, while small varicoceles are demonstrated by performing the Valsalva manoeuvre [4]. Dubin and Amelar's classification, a widely used system, categorises varicoceles into three grades based on visibility and palpability during a physical examination: grade I (small, detectable only during the Valsalva manoeuvre while standing), grade II (moderate, palpated easily without the Valsalva manoeuvre), and grade III (large, causing visible bulging of the scrotal skin, resembling a "bag of worms") [5]. Color Doppler ultrasound is the preferred imaging technique, but the necessity of imaging itself remains debated [6]. Several surgical techniques for managing varicocele aim to eliminate venous blood reflux into the scrotum. These methods include clipping or surgically ligating the varicocele through open, laparoscopic, or microsurgical varicocelectomy, performed at various anatomical levels such as suprainguinal, inguinal, and subinguinal. Additionally, percutaneous embolisation of the gonadal vein and sclerotherapy are other minimally invasive treatment options [7]. Our study aimed to compare surgical outcomes and postoperative complications between the suprainguinal and inguinal approaches for varicocelectomy, contributing to evidence-based decision-making in varicocele management and enhancing patient care and fertility outcomes.

## Materials and methods

The prospective comparative study was carried out at the Department of General Surgery of Dr. D. Y. Patil Medical College, Hospital and Research Centre in Pimpri, Pune, India, from August 2022 to July 2024 following approval from the institution's Institutional Ethics Sub-Committee (approval number: I.E.S.C./341/2022). A total of 60 consenting male patients, aged between 18 and 40 years, diagnosed with grade II or III unilateral or bilateral varicocele and presenting with one of three clinical indications, symptoms (pain), infertility, or fitness concerns, were included in the study. The study excluded patients with subclinical or grade I varicocele, recurrent varicocele, and concomitant inguinoscrotal pathologies such as hydrocele, epididymal cyst, and inguinal hernia.

The patients were randomly assigned to two groups using the chit method. Group A (n=30) underwent the suprainguinal approach for varicocelectomy, while Group B (n=30) underwent the inguinal approach. Preoperative preparation included a detailed history and clinical examination, including the Valsalva manoeuvre, baseline investigations (complete blood picture including hemoglobin and total leucocyte count, liver and renal function tests, serology, coagulation profile, urine analysis, chest X-ray, echocardiogram), ultrasonography of the abdomen, pelvis, and inguinoscrotal region with color Doppler. Preoperative semen analysis was conducted on 12 patients in the infertility group (five in Group A and seven in Group B). All operations were carried out under spinal anaesthesia. In Group A, a transverse incision of 4-5 cm was made at the level of the anterior superior iliac spine along the mid-inguinal line. The subcutaneous tissue and the external, internal oblique, and transverse muscles were dissected to expose the peritoneum, which was subsequently pushed medially. The testicular veins were isolated and ligated after being separated from the testicular artery, and the wound was closed in layers. In Group B, a skin incision was made 1 cm above and medial to the inguinal ligament. The subcutaneous tissue and external oblique aponeurosis were incised. The spermatic cord was identified following the opening of the spermatic fascia. The dilated and tortuous veins were carefully dissected from the vas deferens and testicular artery and then ligated and excised. The wound was subsequently closed in layers.

Postoperative care included intravenous fluids, antibiotics (third-generation cephalosporins), and analgesics on the day of the operation. Postoperative findings were recorded, and all patients were monitored during follow-up visits at one, three, and six months. Semen analyses were performed six months postoperatively on patients in the infertility group.

Statistical analysis

Descriptive statistics, such as means, ranges, and standard deviations (SD), were used to summarise continuous variables like operating time and postoperative pain scores. Inferential statistics, such as chi-squared tests for categorical variables, were utilised to determine if there are significant differences in the two surgical approaches regarding postoperative complications. All data were compiled into an Excel spreadsheet and analysed using the SPSS software, with a p-value of <0.05 indicating statistical significance.

## Results

A total of 60 patients participated in the study. The mean (±SD) age of the study population was 29.05±5.96 years, ranging from 18 to 40 years. Of these patients, 65% (n=39) had left-sided varicoceles, 26.6% (n=16) had right-sided varicoceles, and 8.3% (n=5) had bilateral varicoceles. Additionally, 61.6% (n=37) had grade II varicocele, while 38.3% (n=23) had grade III varicocele. Among the patients, 70% (n=42) were smokers, and 30% (n=18) were non-smokers. Clinical indications for presentation included pain in 70% (n=42) of patients, infertility in 20% (n=12), and fitness concerns in 10% (n=6) (Table 1).

**Table 1:** Demographic and clinical characteristics of the study population (n=60) Data presented as n (%); n denotes the frequency

Characteristics		n (%)
Age group (in years)	18-25	21 (35)
	26-30	14 (23.3)
	31-35	14 (23.3)
	36-40	11 (18.3)
Laterality	Left	39 (65)
	Right	16 (26.6)
	Bilateral	5 (8.3)
Grade of varicocele	II	37 (61.6)
	III	23 (38.3)
Clinical presentation	Pain	42 (70)
	Infertility	12 (20)
	Fitness	6 (10)

The operating time in Group A was significantly shorter as compared to Group B (33.1 vs. 40.8 minutes, p<0.001). Postoperative pain was assessed using a Visual Analogue Scale (VAS), and the mean number of analgesics consumed per week showed comparable results for both groups. However, the difference was not statistically significant (Table 2).

**Table 2:** Surgical outcomes between suprainguinal and inguinal approaches (n=60) Data presented as mean (±SD) with p-value by chi-squared test; *p-value <0.05 considered statistically significant; n denotes the frequency VAS: Visual Analogue Scale

Parameter	Suprainguinal (n=30)	Inguinal (n=30)	P-value
Operating time (minutes)	33.1±6.0	40.8±3.5	<0.001*
Postoperative pain (VAS)	5.3±1.3	5.0±1.3	0.421
Number of analgesics consumed/week	7.7±2.4	7.3±2.1	0.361
Improvement in semen parameters	4	5	0.735

Out of 12 patients, nine (75%) showed improvement in semen parameters postoperatively for both approaches. The improvement in this parameter was independent of the surgical approach, with Groups A and B showing four (80%) and five (71.4%) improvement cases, respectively, and no statistically significant difference between the groups (p=0.735) (Table 2). As the total number was only 12 in the infertile group, it was impossible to assess the relative supremacy in this regard.

The incidence of wound hematoma, wound infection, and hydrocele development was comparable between the groups. Specifically, hematoma occurred in three patients (one in Group A and two in Group B), wound infection occurred in six patients (two in Group A and four in Group B), and hydrocele development was noted in three patients (one in Group A and two in Group B), with statistical insignificance (p-values of 0.554, 0.389, and 0.554, respectively) (Table 3). No patients with testicular atrophy were observed in either group.

**Table 3:** Summary of complications between suprainguinal and inguinal approaches Data presented as n (%) with p-value by chi-squared test; p-value <0.05 considered statistically significant; n denotes the frequency

Parameter	Suprainguinal (n=30)	Inguinal (n=30)	Total (n=60)	P-value
Wound hematoma	1 (3.3)	2 (6.6)	3 (5)	0.554
Wound infection	2 (6.6)	4 (13.3)	6 (10)	0.389
Development of hydrocele	1 (3.3)	2 (6.6)	3 (5)	0.554

**Figure 1:**
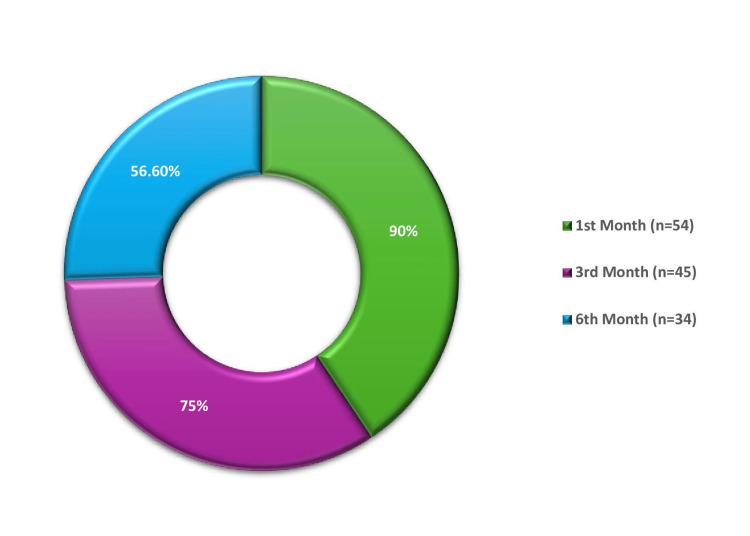
Follow-up profile of 60 patients Data presented as percentage

The follow-up compliance in our setting needs to be revised. In the first month, 54 out of 60 patients (90%) turned up. By the third month, the number of patients available for follow-up decreased to 45 (75%), and in the sixth month, only 34 patients (56.6%) turned up. However, the infertility group had a relatively higher follow-up rate at six months, with 10 out of 12 patients (83.3% of the group) returning for follow-up (Figure 1).

## Discussion

Varicocele can affect individuals of any age, with documented cases even as young as 18 months old. Our study included a diverse age distribution among participants, with the predominant group being 18-25 years old, representing 21 patients (35%) of the total sample and a mean age of 29.05±5.96. Left-sided varicocele is the most frequently observed condition in clinical practice [2]. In our study, 39 patients (65%) had left-sided varicoceles, 16 (26.6%) had right-sided varicoceles, and five (8.3%) had bilateral varicoceles. Similarly, Ahmed et al. reported that varicocele was observed in 65.7% of cases on the left, 7.8% on the right, and 26.3% bilaterally [8]. These proportions are consistent with those reported in other studies [5,9-11].

In our study, 42 patients (70%) presented with a dragging type of pain, 12 (20%) with infertility, and six (10%) for recruitment in the armed forces or government jobs. Ahmed et al. and Arain et al. also found that a dragging sensation was the most common complaint, with 65.79% and 53.9%, respectively [8,9]. Kaye and Omar et al. reported findings that were consistent with our study [12,13].

The duration for varicocelectomy via the suprainguinal approach was found to be slightly shorter than the inguinal approach (33.1 vs. 40.8 minutes), aligning with the observations of the study by Hameed-Ur-Rahman et al., which also reported a shorter duration for the suprainguinal approach [14]. This difference in operating times could be attributed to the more direct access to the spermatic vessels, which allows for quicker identification and ligation.

Postoperative pain, as measured by the VAS and postoperative consumption of analgesics per week, showed no statistically significant difference between the two approaches. Similar results by Omar et al. and Hameed-Ur-Rahman et al. also observed comparable results between the two approaches and could not determine relative supremacy [13,14].

Regarding complications, in our study, hematoma formation was observed in two patients (6.67%) in the inguinal approach group and one (3.34%) in the suprainguinal approach group, likely due to the increased manipulation of the cord structures in the inguinal approach. The incidence of wound infection was one (3.34%) in the inguinal approach group, while there were no cases in the suprainguinal approach group, which is also statistically insignificant. Ahmed et al. found a hematoma incidence of 13.16% in the inguinal group and 2.63% in the suprainguinal group, with infection rates of 7.89% in the inguinal group and 2.63% in the suprainguinal group [8]. Similarly, Arain et al. noted a hematoma incidence of 7.7% in the inguinal group and 3.9% in the suprainguinal group, with a wound infection rate of 3.9% in the inguinal group and none in the suprainguinal group [9]. Both studies indicated statistically insignificant p-values and are consistent with our findings. Similarly, Omar et al. reported a 5% incidence of wound infection and a 10% incidence of hematoma formation in the inguinal group. In contrast, the retroperitoneal group had no cases of wound infection or hematoma [13].

The postoperative development of hydrocele is a common complication resulting from the disruption of lymphatic vessels, with an incidence ranging from 3% to 39% in the literature [15]. However, in our study, hydrocele formation was observed in two patients (6.67%) in the inguinal approach group and one (3.34%) in the suprainguinal group. This may be due to the limited follow-up duration and poor patient compliance. In similar studies conducted by Ahmed et al., Omar et al., and Arain et al., hydrocele incidence was found to be 13.16%, 5%, and 3.9%, respectively, in patients undergoing the inguinal approach to varicocele surgery. In contrast, no cases of hydrocele were detected in those undergoing the suprainguinal approach, highlighting it as the better approach [8,9,13].

Our study observed no postoperative testicular atrophy in either group because of the meticulous preservation of the testicular artery. However, the inguinal approach had a 1% incidence, which can also be avoided using microsurgical loupes and Doppler intraoperatively [16]. The findings align with those of Arain et al. and Omar et al. In contrast, Ahmed et al. observed a 13.1% incidence of testicular atrophy in the inguinal group and no cases in the suprainguinal group [8,9,13].

Overall, these findings indicate that while there are slight differences in complication rates between the suprainguinal and inguinal approaches, none are statistically significant, indicating comparable safety profiles.

Following varicocelectomy, semen morphology typically shows gradual improvement in the postoperative period. In our study, nine out of 12 patients (75%) experienced improved semen parameters based on morphology, motility, and spermatozoa count. Various studies have found that 40-70% of patients undergoing varicocele surgery experience significant improvements in semen quality and fertility outcomes. However, the results did not show a preference for any specific surgical approach regarding improvements in semen quality. In concordance with findings from Hameed-Ur-Rahman et al., Ahmed et al. reported a 90% improvement in semen quality post-surgery. In comparison, Arain et al. observed a 100% improvement in semen morphology nine months and one year after surgery [8,9,14]. These findings were further supported by studies from Al-Said et al. and Chiba and Fujisawa [17,18].

Further research with a larger sample size and careful assessment of long-term complications, such as varicocele recurrence or late-onset testicular atrophy, is essential for determining the optimal approach to managing varicocele and significantly enhancing the existing knowledge base on this condition.

## Conclusions

The suprainguinal and inguinal approaches to varicocelectomy are widely used surgical methods, each with distinct advantages and drawbacks. However, notable distinctions exist that may influence the choice of approach. The suprainguinal approach may be preferable in clinical situations where a shorter operating time is crucial, as it is simpler and easier to perform, avoiding disruption of the inguinal canal anatomy and encountering fewer vein divisions. In the current study, postoperative complications and improvements in semen parameters were comparable between the two methods. Both procedures effectively improve semen parameters, irrespective of the surgical approach. Therefore, it is essential to thoroughly evaluate all patients presenting with infertility for the presence of varicocele, and surgical intervention should be considered for all diagnosed cases.
